# Safety Monitoring of Pfizer-BioNTech COVID-19 Vaccine Booster Doses Among Children Aged 5–11 Years — United States, May 17–July 31, 2022

**DOI:** 10.15585/mmwr.mm7133a3

**Published:** 2022-08-19

**Authors:** Anne M. Hause, James Baggs, Paige Marquez, Tanya R. Myers, John R. Su, Brandon Hugueley, Deborah Thompson, Julianne Gee, Tom T. Shimabukuro, David K. Shay

**Affiliations:** ^1^CDC COVID-19 Emergency Response Team; ^2^Food and Drug Administration, Silver Spring, Maryland.

On May 17, 2022, the Food and Drug Administration (FDA) amended the Emergency Use Authorization (EUA) for BNT162b2 (Pfizer-BioNTech) COVID-19 vaccine to authorize a homologous* booster dose for children aged 5–11 years ≥5 months after receipt of the second primary series dose^†^ (*1*) based on findings from a clinical trial conducted among 401 children aged 5–11 years (*2*). To further characterize the safety of booster vaccination in this age group, CDC reviewed adverse events and health impact assessments after receipt of a Pfizer-BioNTech third dose reported to v-safe, a voluntary smartphone-based safety surveillance system for adverse events occurring after COVID-19 vaccination, and adverse events reported to the Vaccine Adverse Event Reporting System (VAERS), a passive vaccine safety surveillance system comanaged by CDC and FDA. During May 17–July 31, 2022, approximately 657,302 U.S. children aged 5–11 years received a third Pfizer-BioNTech dose (either a third primary series dose administered to immunocompromised children or a booster dose administered to immunocompetent children)^§^; 3,249 Pfizer-BioNTech third doses were reported to v-safe for children in this age group. Local and systemic reactions were reported to v-safe after a second dose and a third dose with similar frequency; some reactions (e.g., pain) were reported to be moderate or severe more frequently after a third dose. VAERS received 581 reports of adverse events after receipt of a Pfizer-BioNTech third dose by children aged 5–11 years; 578 (99.5%) reports were considered nonserious, and the most common events reported were vaccine administration errors. Three (0.5%) reports were considered serious; no reports of myocarditis or death were received. Local and systemic reactions were common among children after Pfizer-BioNTech third dose vaccination, but reports of serious adverse events were rare. Initial safety findings are consistent with those of the clinical trial (*2*).

V-safe is a voluntary smartphone-based U.S. active safety surveillance system established to monitor adverse events after COVID-19 vaccination (https://vsafe.cdc.gov/en/). The v-safe platform allows existing registrants to report receipt of a third COVID-19 vaccine dose and new registrants to enter information about all doses they received. Registrants aged ≤15 years must be enrolled by a parent or guardian. Health surveys are sent daily during the first week after vaccine administration and include questions about potential local injection site and systemic reactions and health impacts.^¶^ CDC’s v-safe call center contacts parents who indicate that medical care was sought for their child after vaccination and encourages completion of a VAERS report, if indicated.

VAERS is a U.S. national passive vaccine safety surveillance system comanaged by CDC and FDA that monitors adverse events after vaccination (*3*). VAERS accepts reports from health care providers, vaccine manufacturers, and members of the public.** VAERS reports are classified as serious if any of the following are reported: hospitalization, prolongation of hospitalization, life-threatening illness, permanent disability, congenital anomaly or birth defect, or death.^††^ VAERS staff members assign Medical Dictionary for Regulatory Activities (MedDRA) preferred terms (PTs) to the signs, symptoms, and diagnostic findings in VAERS reports.^§§^ CDC and FDA physicians reviewed serious reports to VAERS to form a clinical impression based on available data. Selected MedDRA PTs were used to search for possible cases of myocarditis. Myocarditis and pericarditis are rare adverse events that have been associated with receipt of mRNA-based COVID-19 vaccines, especially among adolescent males and young adults (*4*).

This report assessed local and systemic reactions and health impacts reported in the week after vaccination among v-safe registrants aged 5–11 years who received a homologous Pfizer-BioNTech third dose ≥5 months after completion of their primary series during May 17–July 31, 2022. At least one survey after a third and at least one survey after a previous vaccine dose were required for inclusion. The odds of reporting an adverse reaction or health impact after receipt of the third dose and previous doses were compared using multivariable generalized estimating equations models.^¶¶^ VAERS reports for children aged 5–11 years who received a Pfizer-BioNTech third dose during May 17–July 31, 2022, were described by serious and nonserious classification, demographic characteristics, and MedDRA PTs.*** SAS software (version 9.4; SAS Institute) was used for all analyses. These surveillance activities were reviewed by CDC and conducted consistent with applicable federal law and CDC policy.^†††^

## Review of v-safe Data

During May 17–July 31, 2022, a total of 3,249 homologous Pfizer-BioNTech third doses were reported to v-safe for children aged 5–11 years. The most frequently reported adverse reactions were injection site pain (2,166; 66.7%), fatigue (938; 28.9%), and headache (647; 19.9%) (Table 1). Most reported reactions were mild in severity; reporting was most frequent the day after vaccination. Local injection site reactions (2,224; 68.5%) and systemic reactions (1,483; 45.6%) were frequently reported after third dose vaccination (Table 2). Local injection site reactions were reported with equal frequency after dose 3 (68.5%) and dose 2 (68.0%) (p = 0.65). The prevalences of reported systemic reactions were similar after dose 3 (45.6%) and dose 2 (45.8%) (p = 0.91). Although mild symptoms were most frequently reported, the frequency of reporting moderate or severe symptoms was higher after receipt of dose 3 than after dose 2 among those reporting pain, fatigue, headache, or myalgia.

**TABLE 1:** Most frequently reported adverse reactions reported* to v-safe for children aged 5–11 years who received homologous Pfizer-BioNTech COVID-19 booster vaccination**^†^** (N = 3,249), by severity**^§^** and dose — United States, May 17–July 31, 2022

Reported event	% Reporting event		
	Dose 1	Dose 2	Dose 3
**Injection site pain**	**60.7**	**66.1**	**66.7**
Mild	50.1	50.7	44.9
Moderate	10.2	14.9	20.8
Severe	0.3	0.6	1.0
**Fatigue**	**22.9**	**29.9**	**28.9**
Mild	15.0	17.5	15.1
Moderate	7.2	11.6	12.0
Severe	0.7	0.8	1.7
**Headache**	**15.2**	**20.6**	**19.9**
Mild	10.5	13.1	11.4
Moderate	4.4	7.1	7.5
Severe	0.2	0.4	1.0
**Myalgia**	**7.1**	**10.2**	**13.9**
Mild	4.8	6.0	7.2
Moderate	2.1	4.0	6.3
Severe	0.2	0.2	0.4
**Chills**	**3.8**	**7.6**	**7.4**
Mild	2.6	4.6	4.1
Moderate	1.1	3.0	2.9
Severe	0.1	0.1	0.4
**Fever^¶^**	**1.4**	**3.9**	**5.1**
Mild	0.9	2.2	2.7
Moderate	0.4	1.0	1.4
Severe	0.1	0.6	0.9
Very severe	0.03	0.1	0.1

* Percentage of registrants who reported a reaction or health impact at least once during days 0–7 after vaccination.

^†^ Includes only persons who received Pfizer-BioNTech COVID-19 vaccine for primary series and first booster dose and completed at least one survey after their booster dose and at least one survey after a previous vaccine dose.

^§^ Includes the most severe episode reported during the day 0–7 window for each event. Parents and guardians who participate in v-safe use the following definitions to describe the severity of a child’s symptoms: mild (noticeable, but not problematic), moderate (limit normal daily activities), or severe (make daily activities difficult or impossible). The odds of reporting a moderate or severe symptom after booster dose and previous doses were compared using a multivariable generalized estimating equations model that accounted for repeated measures among doses reported by each registrant; statistical significance was defined by p<0.05. All booster dose and dose 1 comparisons were statistically significant (p<0.01). All booster dose and dose 2 comparisons were statistically significant (p<0.05) except “chills” (p = 0.38).

^¶^ Includes those who reported a temperature and met the definition for fever (≥100.4°F [≥38.0°C]) during days 0–3. If information was available, fever was classified further as mild (100.4°F–101.1°F [38.0°C–38.3°C]), moderate (101.2°F–102.0°F [38.4°C–38.9°C]), severe (102.1°F–104.0°F [39.0°C–40.0°C]), or very severe (>104.0°F [>40°C]). Because few registrants reported a temperature that met the definition for fever, statistics were not estimated for this variable.

**TABLE 2:** Adverse reactions and health impacts reported to v-safe for children aged 5–11 years who received homologous Pfizer-BioNTech COVID-19 booster vaccination* (N = 3,249) — United States, May 17–July 31, 2022

Reported event	% Reporting event^†^		
	Dose 1	Dose 2	Dose 3
**Any local injection site reaction**	**62.6**	**68.0**	**68.5**
Itching	4.9	4.9	5.3
Pain	60.7	66.1	66.7
Redness	4.5	5.5	8.5
Swelling	4.2	6.2	9.6
**Any systemic reaction**	**38.1**	**45.8**	**45.6**
Abdominal pain	5.3	7.4	6.1
Myalgia	7.1	10.2	13.9
Chills	3.8	7.6	7.4
Diarrhea	2.6	2.2	2.4
Fatigue	22.9	29.9	28.9
Fever	7.8	15.4	16.9
Headache	15.2	20.6	19.9
Joint pain	2.2	3.0	3.4
Nausea	4.8	7.1	7.1
Rash	1.0	0.8	1.3
Vomiting	1.9	2.5	3.1
**Any health impact**	**9.4**	**14.5**	**16.3**
Unable to perform normal daily activities	4.7	7.5	12.1
Unable to attend school	6.5	10.0	6.9
Needed medical care	1.1	0.9	1.0
Clinic	0.5	0.5	0.5
Telehealth	0.2	0.2	0.3
Emergency department visit	0.03	0.1	0.03
Hospitalization	0.03	0	0

* Includes only persons who received Pfizer-BioNTech COVID-19 vaccine for primary series and first booster dose and completed at least one survey after their booster dose and at least one survey after a previous vaccine dose.

^†^ Percentage of registrants who reported a reaction or health impact at least once during days 0–7 after vaccination. The odds of reporting any local injection site or systemic reaction or health impact after booster dose and previous doses were compared using a multivariable generalized estimating equations model that accounted for repeated measures among doses reported by each registrant; the threshold for statistical significance was p<0.05. All booster dose and dose 1 comparisons were statistically significant (p<0.001), except “unable to attend school” and “needed medical care.” Among booster dose and dose 2 comparisons, “any health impact” (p<0.05), “unable to perform normal daily activities” (p<0.001), and “unable to attend school” (p<0.001) were statistically significant; “needed medical care” was not significantly different.

In the week after third dose vaccination, 6.9% (225) of enrolled children were reported to be unable to attend school, and 12.1% (392) were unable to complete daily activities. Approximately 1.0% of parents reported seeking medical care for their child after third dose vaccination, most commonly in an outpatient clinic (16; 0.5%) or via telehealth visit (11; 0.3%). No children received care at a hospital after third dose vaccination. Inability to attend school was reported less frequently after receipt of dose 3 (6.9%) than after dose 2 (10.0%) (p<0.001). Inability to complete daily activities was reported more frequently after dose 3 (12.1%) than after dose 2 (7.5%) (p<0.001). Receipt of medical care after dose 3 (1.0%) and dose 2 (0.9%) did not differ significantly (p = 0.52).

## Review of VAERS Data

During May 17–July 31, 2022, VAERS received and processed 581 reports of one or more adverse events after Pfizer-BioNTech third dose vaccination among children aged 5–11 years; recipients’ median age was 9 years, and 275 (47.3%) reports were for girls. Most reports (573; 98.6%) indicated that the third COVID-19 dose was the sole vaccine administered at the encounter. Overall, 578 (99.5%) VAERS reports were classified as nonserious (Table 3). Among nonserious reports, the most commonly reported events (413; 71.1%) were related to vaccine preparation or administration errors (e.g., product preparation issue or error, incorrect dose administered, and product administered to patient of inappropriate age); 63 (15.3%) of these 413 reports also listed an adverse health event. Other commonly reported events among nonserious reports included fever (45; 7.8%), pain in extremity (38; 6.6%), and fatigue (28; 4.8%). The three serious reports included new onset type 1 diabetes 10 days after vaccination, facial swelling 3 days after vaccination, and generalized pain, fatigue, and malaise 5 days after vaccination requiring hospitalization. There were no reports to VAERS of either myocarditis or death.

**TABLE 3:** Reports of nonserious and serious events to the Vaccine Adverse Event Reporting System for children aged 5–11 years who received a Pfizer-BioNTech COVID-19 booster dose (N = 581) — United States, May 17–July 31, 2022

Reported events	No. (%)
**Nonserious VAERS reports**	578 (100)
Symptom, sign, diagnostic result, or condition (MedDRA PT*)	
Product preparation issue	145 (25.1)
Incorrect dose administered	128 (22.2)
No adverse event^†^	105 (18.2)
Product administered to patient of inappropriate age	55 (9.5)
Product preparation error	53 (9.2)
Expired product administered	46 (8.0)
Fever	45 (7.8)
Pain in extremity	38 (6.6)
Fatigue	28 (4.8)
Headache	22 (3.8)
Injection site pain	22 (3.8)
Product storage error	22 (3.8)
Vomiting	22 (3.8)
Chills	18 (3.1)
Dizziness	18 (3.1)
**Serious VAERS reports** ^§,¶^	**3 (100)**
**Clinical impression**	
Generalized pain, fatigue, and malaise requiring hospitalization	1 (33.3)
New onset type 1 diabetes	1 (33.3)
Facial swelling	1 (33.3)

**Abbreviations**: MedDRA PT = Medical Dictionary for Regulatory Activities preferred terms; VAERS = Vaccine Adverse Event Reporting System.

* Signs and symptoms in VAERS reports are assigned MedDRA PTs by VAERS staff members. Each VAERS report might be assigned more than one MedDRA PT and can include normal diagnostic findings. A MedDRA PT does not represent a medical diagnosis made or confirmed by a provider or clinical reviewer.

^†^ All reports classified as no adverse event were accompanied by at least one report of vaccine error (e.g., product preparation issue, incorrect dose administered, product preparation error, product administered to patient of inappropriate age, expired product administered, or product storage error). A total of 413 reports were classified as vaccine errors; the most common specific errors are listed in the table. Of the 413 reports of vaccine error, 105 included the MedDRA PT “no adverse event,” 63 listed an adverse health event, and the remaining reports only indicated that a vaccine error occurred.

^§^ VAERS reports are classified as serious if any of the following are reported: hospitalization, prolongation of hospitalization, life-threatening illness, permanent disability, congenital anomaly or birth defect, or death.

^¶^ Serious reports to VAERS were reviewed by CDC physicians to form a clinical impression. The clinical impression of the event does not establish a causal role with vaccination. https://www.meddra.org/how-to-use/basics/hierarchy

## Discussion

This report provides safety findings from v-safe and VAERS data collected during the first 10 weeks of administration of Pfizer-BioNTech booster doses to children aged 5–11 years, a period in which approximately 657,302 third doses were administered in this age group. Adverse reactions reported to v-safe and VAERS for children aged 5–11 years after receipt of a third dose were similar to adverse reactions reported in the Pfizer-BioNTech clinical trial, reinforcing the safety of vaccination in this population (*2*).

Among reports to v-safe for children aged 5–11 years, reports of local and systemic reactions after third dose vaccination were similar in frequency to those reported after a primary series (*5*–*7*). Although local and systemic reactions were similarly reported after receipt of dose 2 and dose 3, some reactions were more frequently reported as moderate or severe after a third than a second dose. This reporting pattern is consistent with clinical trial results (*2*). Parents reported symptom severity in v-safe based on how the symptom affected their child’s ability to complete daily activities. Thus, more common reporting of moderate-to-severe reactions likely reflects increased reporting of the health impact “inability to perform normal daily activities.” However, there was no significant difference between the proportions of children receiving medical attention after receipt of the second or third doses of Pfizer-BioNTech vaccine.

Approximately 99% of reports to VAERS for children aged 5–11 years after a Pfizer-BioNTech third dose were classified as nonserious. The most common adverse events reported were related to vaccine administration errors, most of which did not have an accompanying adverse health event. Children aged 5–11 years were the first to receive a smaller amount of mRNA (10 *μ*g, 0.2 mL) than that recommended for persons aged ≥12 years (30 *μ*g, 0.3 mL) (*1*). Therefore, continued education of vaccine providers might help reduce administration errors, including incorrect dosing, among children. Other common reactions reflect known associations with mRNA vaccines. These findings are consistent with previous analyses of VAERS reports following primary series vaccination in this age group (*5*,*6*).

No VAERS reports of myocarditis after third doses among children aged 5–11 years were received. Among children and adolescents aged <18 years, myocarditis risk after COVID-19 vaccination is higher in males (*4*), and risk decreases with decreasing age (*4*,*8*); the myocarditis reporting rate to VAERS after dose 2 was 2.6 per 1 million doses among boys aged 5–11 years and 46.4 per 1 million doses among males aged 12–15 years (*8*). The risk for myocarditis after dose 3 appears to be less than that after dose 2; among males aged 12–15 years, the reporting rate to VAERS after dose 3 (15.3 per 1 million doses) was approximately one third of that after dose 2 (46.4) (*8*).

The findings in this report are subject to at least five limitations. First, v-safe participation is voluntary, and data might not be representative of the entire vaccinated U.S. population. Second, recipients who experience an adverse event might be more likely to respond to v-safe surveys. Third, v-safe does not include information about immune status; third dose recipients likely include persons with and without immunocompromising conditions. Fourth, VAERS is subject to reporting biases and underreporting, especially of nonserious events (*3*). Finally, these data are limited by the 10-week surveillance period. Findings might change as safety monitoring continues and more children aged 5–11 years receive booster doses. In particular, the frequency of vaccine error reports might decline as vaccine administrators gain additional experience with pediatric doses of mRNA COVID-19 vaccines.

The Advisory Committee on Immunization Practices recommends that all children aged 5–11 years receive 1 COVID-19 mRNA booster dose ≥5 months after completion of their primary COVID-19 mRNA series; immunocompromised children aged 5–11 years are recommended to receive a 3-dose primary series (with dose 3 administered ≥4 weeks after dose 2), followed by a booster dose ≥3 months after completion of the primary series.^§§§^ Vaccination continues to be the most effective preventive measure against serious illness and death from COVID-19. Preliminary safety findings for third doses administered to children aged 5–11 years are generally similar to those reported in the clinical trial (*2*). Health care providers and parents should expect local and systemic reactions among children in the week after Pfizer-BioNTech booster vaccination. Serious reports of adverse events are rare. CDC and FDA will continue to monitor vaccine safety and will provide updates as needed to guide COVID-19 vaccination recommendations.

**Summary**

**What is already known about this topic?**

A Pfizer-BioNTech COVID-19 vaccine booster dose is recommended for children aged 5–11 years; approximately 657,302 third doses were administered to children in this age group during May–July 2022.

**What is added by this report?**

Among children aged 5–11 years, local and systemic reactions were reported to v-safe with similar frequency after doses 2 and 3; specific reactions differed in severity. Vaccine administration errors were the most common events reported to the Vaccine Adverse Event Reporting System. No reports of myocarditis or death after receipt of dose 3 were received.

**What are the implications for public health practice?**

Among children aged 5–11 years, serious adverse events after dose 3 are rare. Additional provider education might prevent vaccine administration errors.

## Footnotes

* Homologous refers to a booster dose of the same product administered for the primary series.

† The Advisory Committee on Immunization Practices recommends that all persons aged ≥5 years receive 1 booster dose of a COVID-19 vaccine ≥5 months after completing their primary series with either Pfizer-BioNTech or Moderna mRNA primary series. At the time of publication, only Pfizer-BioNTech vaccine was authorized for use as a booster dose among children aged 5–17 years. https://www.cdc.gov/vaccines/covid-19/clinical-considerations/covid-19-vaccines-us.html

§
https://covid.cdc.gov/covid-data-tracker/#vaccination-demographics-trends

¶ Health surveys for the most recent dose reported are sent via text messages that link to web-based surveys on days 0–7 after receipt of a vaccine dose, then weekly during 6 weeks after vaccination, and then at 3, 6, and 12 months after vaccination. Local injection site reactions include itching, pain, redness, and swelling. Systemic reactions include abdominal pain, myalgia, chills, diarrhea, fatigue, fever, headache, joint pain, nausea, rash, and vomiting. Health impacts include inability to perform normal daily activities, inability to attend school, and receipt of medical care. Parents and guardians use the following definitions to describe the severity of a child’s symptoms: mild (noticeable, but not problematic), moderate (limit normal daily activities), or severe (make daily activities difficult or impossible).

** CDC and FDA encourage health care providers to report adverse events to VAERS, and providers are required by COVID-19 vaccine EUAs to report certain adverse events, including death, after vaccination to VAERS. https://vaers.hhs.gov/faq.html

†† VAERS reports are classified as serious based on 21 C.F.R. https://www.accessdata.fda.gov/scripts/cdrh/cfdocs/cfcfr/cfrsearch.cfm?fr

§§ Each VAERS report might be assigned more than one MedDRA PT. A MedDRA-coded event does not indicate a medically confirmed diagnosis. https://www.meddra.org/how-to-use/basics/hierarchy

¶¶ This model accounted for repeated measures among doses reported by each registrant. The threshold for statistical significance was p<0.05.

*** This analysis excluded reports to v-safe or VAERS of children aged 5–11 years who were vaccinated with a booster dose before authorization for a booster dose for their age group (i.e., before May 17, 2022).

††† 45 C.F.R. part 46, 21 C.F.R. part 56; 42 U.S.C. Sect. 241(d); 5 U.S.C. Sect. 552a; 44 U.S.C. Sect. 3501 et seq.

§§§
https://www.cdc.gov/vaccines/acip/recs/grade/pfizer-biontech-covid19-booster-children-etr.html
